# Usp22 deficiency impairs intestinal epithelial lineage specification *in vivo*

**DOI:** 10.18632/oncotarget.5412

**Published:** 2015-09-25

**Authors:** Robyn L. Kosinsky, Florian Wegwitz, Nicole Hellbach, Matthias Dobbelstein, Ahmed Mansouri, Tanja Vogel, Yvonne Begus-Nahrmann, Steven A. Johnsen

**Affiliations:** ^1^ Department of General, Visceral and Pediatric Surgery, University Medical Center Göttingen, 37075 Göttingen, Germany; ^2^ Institute of Molecular Oncology, Göttingen Center of Molecular Biosciences (GZMB), Faculty of Medicine, University of Göttingen, 37077 Göttingen, Germany; ^3^ Department of Molecular Embryology, Institute of Anatomy and Cell Biology, Faculty of Medicine, University of Freiburg, 79104 Freiburg, Germany; ^4^ Department of Molecular Cell Biology, Max-Planck Institute for Biophysical Chemistry, RG Molecular Cell Differentiation, 37077 Göttingen, Germany

**Keywords:** epigenetics, intestinal tract, cell differentiation

## Abstract

Epigenetic regulatory mechanisms play a central role in controlling gene expression during development, cell differentiation and tumorigenesis. Monoubiquitination of histone H2B is one epigenetic modification which is dynamically regulated by the opposing activities of specific ubiquitin ligases and deubiquitinating enzymes (DUBs). The Ubiquitin-specific Protease 22 (USP22) is the ubiquitin hydrolase component of the human SAGA complex which deubiquitinates histone H2B during transcription. Recently, many studies have investigated an oncogenic potential of USP22 overexpression. However, its physiological function in organ maintenance, development and its cellular function remain largely unknown. A previous study reported embryonic lethality in *Usp22* knockout mice. Here we describe a mouse model with a global reduction of USP22 levels which expresses the LacZ gene under the control of the endogenous *Usp22* promoter. Using this reporter we found Usp22 to be ubiquitously expressed in murine embryos. Notably, adult Usp22^lacZ/lacZ^ displayed low residual *Usp22* expression levels coupled with a reduced body size and weight. Interestingly, the reduction of *Usp22* significantly influenced the frequency of differentiated cells in the small intestine and the brain while H2B and H2Bub1 levels remained constant. Taken together, we provide evidence for a physiological role for USP22 in controlling cell differentiation and lineage specification.

## INTRODUCTION

Proper regulation of gene expression patterns is crucial for embryonic development, cell fate determination and differentiation [1–3]. Differentiation-associated changes in transcription are closely associated with alterations in epigenetic modifications such as methylation, acetylation, phosphorylation and monoubiquitination of histones [4]. In humans the obligate heterodimeric RNF20/RNF40 complex functions as the E3 ubiquitin ligase responsible for the monoubiquitination of histone H2B (H2Bub1) [5]. Previous studies revealed an essential role of RNF20 and RNF40 in controlling the expression of a subset of genes [6, 7] and a particular role in cell differentiation [8, 9]. Ubiquitination is removed from H2B via the deubiquitinating module (mDUB) of the SAGA (Spt–Ada–Gcn5 acetyltransferase) transcriptional coactivator complex. Ubiquitin-specific Protease 22 (USP22) is the deubiquitinating subunit of the SAGA mDUB complex [10] and has been reported to affect transcription by the deubiquitination of histones H2A and H2B [11–14]. Additionally, USP22 functions to stabilize specific proteins via the removal of ubiquitin moieties, thereby preventing proteasomal degradation [15–25]. Notably, in addition to H2B, the histone deacetylase Sirt1 has been reported to be a central deubiquitination target of USP22 [22]. USP22 was also reported to control cell cycle progression since its depletion resulted in the induction of a G1 cell cycle arrest in colorectal cancer cells *in vitro* [26]. Consistent with this effect, USP22 was implicated in the expression of MYC target genes such as Cyclin D1 and its presence is required for MYC-mediated transformation in non-small cell lung carcinoma cells [10].

A number of studies have suggested an important function for USP22 during tumorigenesis and/or tumor progression. Notably, *USP22* was initially reported as a member of an 11-gene “death from cancer” gene expression signature which identified tumors displaying a stem cell-like gene expression profile characterized by high malignancy and metastatic dissemination [27]. Since then USP22 has been reported to stabilize some cancer-associated proteins and increased expression was correlated to poor prognosis [15, 16, 19, 21–23, 25]. In particular, a role of USP22 has been suggested in non-small cell lung cancer [15, 21, 28], gastric carcinoma [29], glioma [30], pancreatic cancer [18, 28], breast [31] and colorectal cancer [25, 32, 33]. While some putative USP22 targets have been identified, the exact molecular mechanisms by which USP22 functions and its physiological role remain to be elucidated.

Initial indications that USP22 plays a role in cell differentiation were suggested by its repressive effect on *SOX2* transcription in ESCs. It could be shown that USP22 is required for differentiation of pluripotent stem cells into all three germ layers [34]. However, to date few studies have examined the role of USP22 *in vivo*. Lee et al. [35] described that *Usp22* is ubiquitously expressed in adult murine tissues and at the early embryonic stages in the midbrain, forebrain, hindbrain and dorsal root ganglia. Genetic ablation of *Usp22* in mice resulted in early embryonal lethality at E10.5 of the post-implantation stage [22].

Here we describe the generation of a mouse model with a highly significant reduction of *Usp22* expression levels. Using β-galactosidase staining we visualized ubiquitous activity of the *Usp22* gene in most embryonic tissues. Adult mice with highly reduced *Usp22* levels are viable but display a growth defect and reduction of body weight. We analyzed the small intestine as this organ represents an attractive model to study epigenetic regulatory processes due to its rapid turnover and its well characterized steps leading to tumorigenesis [36, 37]. While the gross morphology of the small intestine was largely unaffected, detailed analyses suggest differentiation defects in the cells of the small intestine. Moreover, analysis of the brain of animals with reduced *Usp22* expression confirmed a general importance for USP22 in differentiation in multiple organs.

## RESULTS

### Generation of Usp22^lacZ^ mice and expression of *Usp22*

**Figure 1:**
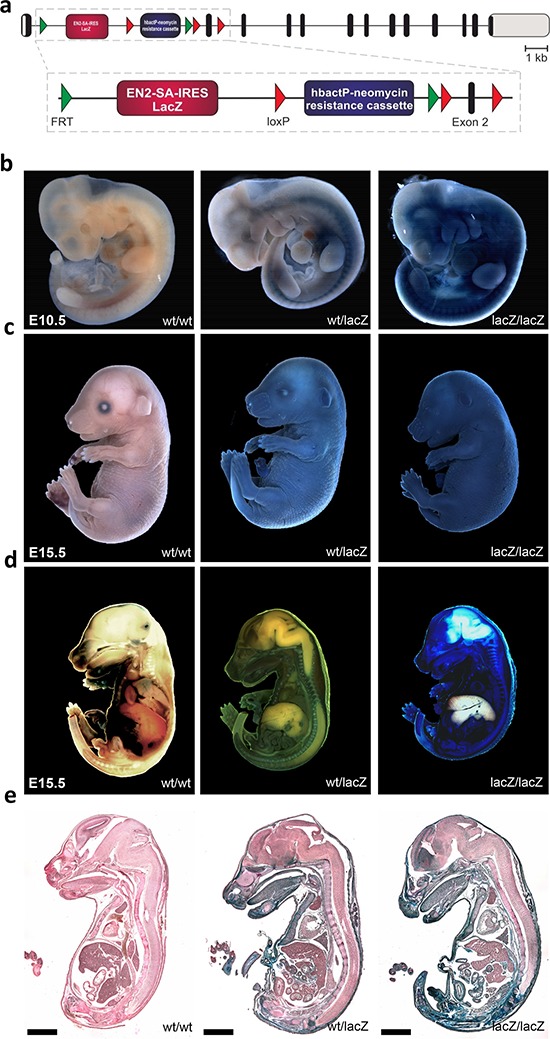
Generation of Usp22^lacZ^ mice and expression of *Usp22* **a.** Schematic overview of the Usp22^lacZ^ mouse construct. The composition of the Usp22^lacZ^ mouse construct is shown with exons indicated as black bars, untranslated regions as grey bars. The LacZ gene coupled to an EN2-SA-IRES sequence is expressed under the control of the endogenous *Usp22* promoter. Selection of successfully recombined fragments is possible due to a neomycin resistance cassette under the control of human β-actin promoter. The decrease of *Usp22* expression is mediated by stop codons and poly-A sites behind the two cassettes in the construct. **b–c.** Usp22^lacZ^ embryos at E10.5 and E15.5 have been stained for the presence of β-galactosidase activity using X-Gal as a substrate. During both developmental stages staining in the skin was observed. **d–e.** The same embryos at E15.5 as in c have been cut on a sagittal plane and stained again for β-galactosidase activity. Subsequently, pictures were taken from the embryos and from 20 μm sections. Paraffin sections have been counterstained. Scale bar: 2000 μm.

In order to characterize both the expression and function of *Usp22*, we generated a mouse line herein referred to as Usp22^LacZ^ using a “knockout first” embryonic stem cell line (Usp22^tm1a(KOMP)Wtsi^ C57Bl6) obtained from the University of California-Davis Knockout Mouse Project Repository. These mice have both a LacZ cassette as well as a neomycin resistance cassette inserted into the first intron of the *Usp22* gene. Due to the presence of a splice acceptor site the LacZ gene is expressed under the control of the endogenous *Usp22* promoter and enables the investigation of *Usp22* gene activity *in vivo* (Figure 1a). Importantly, the resulting allele is predicted to result in the truncation of *Usp22* expression due to the presence of polyadenylation sequences downstream of the LacZ and neomycin resistance genes, while leaving the *Usp22* gene otherwise intact. Mice were backcrossed to the C57Bl6 background.

*Usp22* has been reported to be expressed in several tissues in murine embryos and was shown to be highly present in the brain. However, only specific organs have been analyzed in embryos at E10.5 and E12.5 so far [35]. In order to visualize the expression pattern of *Usp22* in more detail, embryos were processed for whole mount staining making use of the enzymatic activity of β-galactosidase which produces a characteristic blue staining upon cleavage of its substrate X-gal. At E10.5 only Usp22^lacZ/lacZ^ animals showed a strong LacZ staining of the skin at E10.5 (Figure 1b) while during development both Usp22^wt/lacZ^ and Usp22^lacZ/lacZ^ animals displayed strong lacZ expression in the skin (Figure 1c).

In order to investigate *Usp22* expression in the inner organs at E15.5 in later development, embryos were cut sagittally and stained a second time (Figure 1d). Subsequently, paraffin sections were prepared and counterstained (Figure 1e, Table 1). Strong staining was not only detected in the skin but also in connective tissue and muscle. This was also observed in the facial region including tongue, lips and nasal cavity. In the brain, particularly strong staining was seen in the frontal lobe, cerebral cortex, subventricular zone and ganglionic eminences. *Usp22* gene activity was only sporadically observed in cells of the mid- and hindbrain. In addition, the following organs displayed prominent *Usp22* expression: heart, lung, kidneys, penis, thymus, bladder, pancreas, thyroid and intestine. In contrast, the liver did not display any detectable staining. Taken together, X-gal staining revealed ubiquitous *Usp22* expression in most embryonic tissues at E15.5. These findings corroborate the findings of a previous RNA-based study [35] and demonstrate pronounced *Usp22* expression in the cerebral cortex, intestine and other internal organs during embryonic development.

**Table 1:** Usp22-LacZ expression in the developing mouse embryo

	Staining intensity		Staining intensity
Front lobe	+++	Tongue	+++
Cerebellum	++	Thyroid	++
Neopallial cortex	++	Heart	++
Ventricular zone	+	Thymus	++
Midbrain	+	Lung	+++
Hindbrain	+	Liver	−
Olfactory lobe	+++	Intestinal system	+++
Spinal cord	+	Pancreas	++
Root ganglion	+++	Kidneys	+++
Nasal cavity	+++	Bladder	+++
Lips	+++	Penis	+++

− no staining

+ sporadic staining

++ strong staining

+++ very strong staining

### Phenotype of adult Usp22^lacZ^ mice

**Figure 2:**
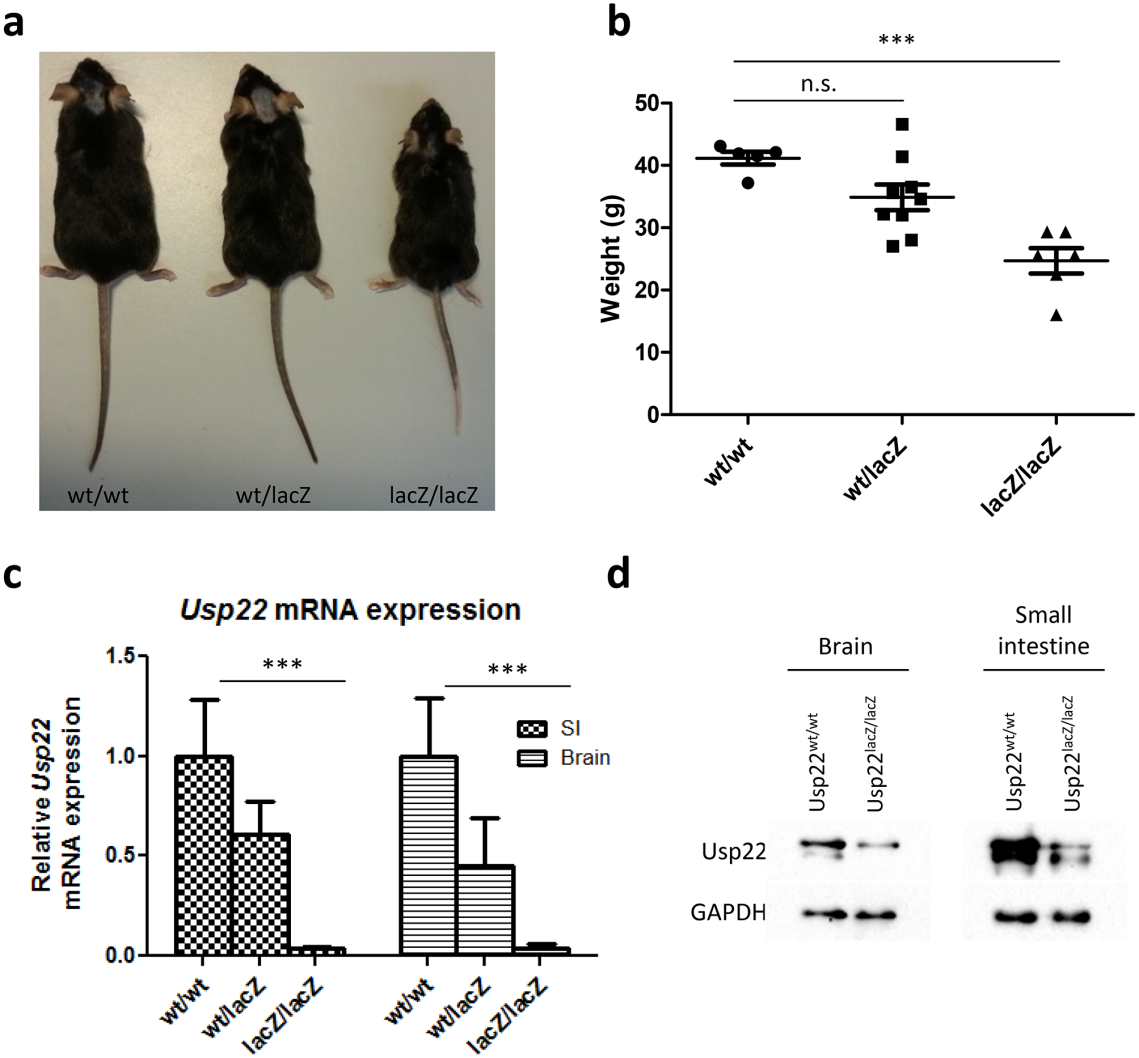
Phenotype of adult Usp22^lacZ^ mice **a.** A representative image of Usp22^lacZ^ animals at an age of 4 months shows growth retardation of Usp22^lacZ/lacZ^ mice. **b.** The body weight of male Usp22^lacZ/lacZ^ mice is reduced on average by 40% compared to their littermates. Mice have been weighed at an age of 4 months (wt/wt: *n* = 5; wt/lacZ: *n* = 9, *p* = 0.525; lacZ/lacZ: *n* = 6, *p* = < 0.0001). **c.** Relative mRNA expression of *Usp22* in the small intestine (wt/wt: *n* = 4; wt/lacZ: *n* = 5, *p* = 0.007; lacZ/lacZ: *n* = 4, *p* = 8.10^−4^). **d.** Reduction of Usp22 protein levels in brain and small intestine of Usp22^lacZ/lacZ^ mice.

In contrast to a previous study reporting that ablation of *Usp22* expression leads to embryonic lethality [22], Usp22^lacZ^ mice were born with the expected Mendelian frequency. For our mouse cohorts Usp22^wt/lacZ^ have been mated with each other. However, one mating with an Usp22^lacZ/lacZ^ female was set up and offspring was obtained suggesting that animals with reduced Usp22 levels are fertile. Interestingly, Usp22^lacZ/lacZ^ mice were, however, characterized by a clear growth retardation compared to their litter mates and their weight was reduced by 40% at the age of 4 months (Figure 2a–2b). Since the intestine reflects an ideal disease-relevant model organ to study epigenetic regulatory mechanisms [36] and our β-galactosidase stainings revealed expression of *Usp22* in both the cerebral cortex and the digestive tract, we aimed to examine the effects of *Usp22* deficiency *in vivo* in both these organs in adult animals. Firstly we studied the expression of *Usp22* in wildtype and Usp22^lacZ/lacZ^ animals. Therefore, the brain and small intestines were isolated from 4 month-old Usp22^lacZ^ mice and *Usp22* expression levels were determined by qRT-PCR. Notably, while *Usp22* expression in both organs of the Usp22^lacZ/lacZ^ mice was significantly reduced, approximately 3% of correctly spliced mRNA levels were observed in mutant animals compared to their wild-type littermates (Figure 2c) indicating that the Usp22^lacZ^ allele is hypomorphic. This was supported by an incomplete reduction of USP22 protein levels in Western blot (Figure 2d).

Collectively, our findings show that, unlike the effects observed with a complete ablation of *Usp22* expression, a significant, but incomplete, reduction of *Usp22* does not result in embryonic lethality, but rather leads to decreased body size and weight compared to their wild-type and heterozygous littermates.

### Decreased *Usp22* expression does not affect small intestine morphology

**Figure 3:**
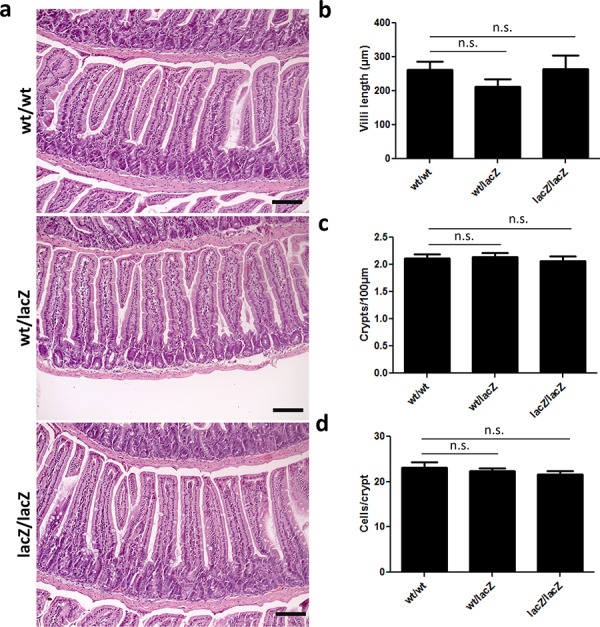
Expression of *Usp22* at the crypt base does not affect small intestine morphology **a–d.** Global reduction of *Usp22* levels does not lead to morphological changes of the small intestine. H&E-stained sections have been assessed regarding to villi length (wt/wt: *n* = 3; wt/lacZ: *n* = 3, *p* = 0.20; lacZ/lacZ: *n* = 3, *p* = 0.98), number of crypts per 100 μm (wt/wt: *n* = 3; wt/lacZ: *n* = 3, *p* = 0.85; lacZ/lacZ: *n* = 4, *p* = 0.65) and corresponding number of cells per crypt (wt/wt: *n* = 3; wt/lacZ: *n* = 3, *p* = 0.63; lacZ/lacZ: *n* = 3, *p* = 0.34). Regarding the morphology no significant differences were detected. Scale bar: 100 μm.

To investigate the role of USP22 in the small intestine we determined the effects of reduced *Usp22* expression on the gross morphology and cellular content of the small intestine. Small intestine sections were stained with H&E and used for subsequent analyses. During all histological studies we carefully assessed proximal, intermediate as well as distal regions of the small intestine. Initially, villi length was measured and the mean values were calculated. When comparing Usp22^wt/wt^ and Usp22^lacZ/lacZ^ animals the mean length per villus was between 264 μm and 266 μm. We next counted approximately 2 crypts within a distance of 100 μm invariantly in all genotypes. Furthermore, the number of cells per crypt was between 22 to 24 cells per crypt as estimated in at least 50 crypts per mouse (Figure 3a–3d). Taken together, these results revealed that the reduction of *Usp22* expression does not alter the gross morphology of the small intestine.

### The loss of *Usp22* results in altered cellular composition in the small intestine

**Figure 4:**
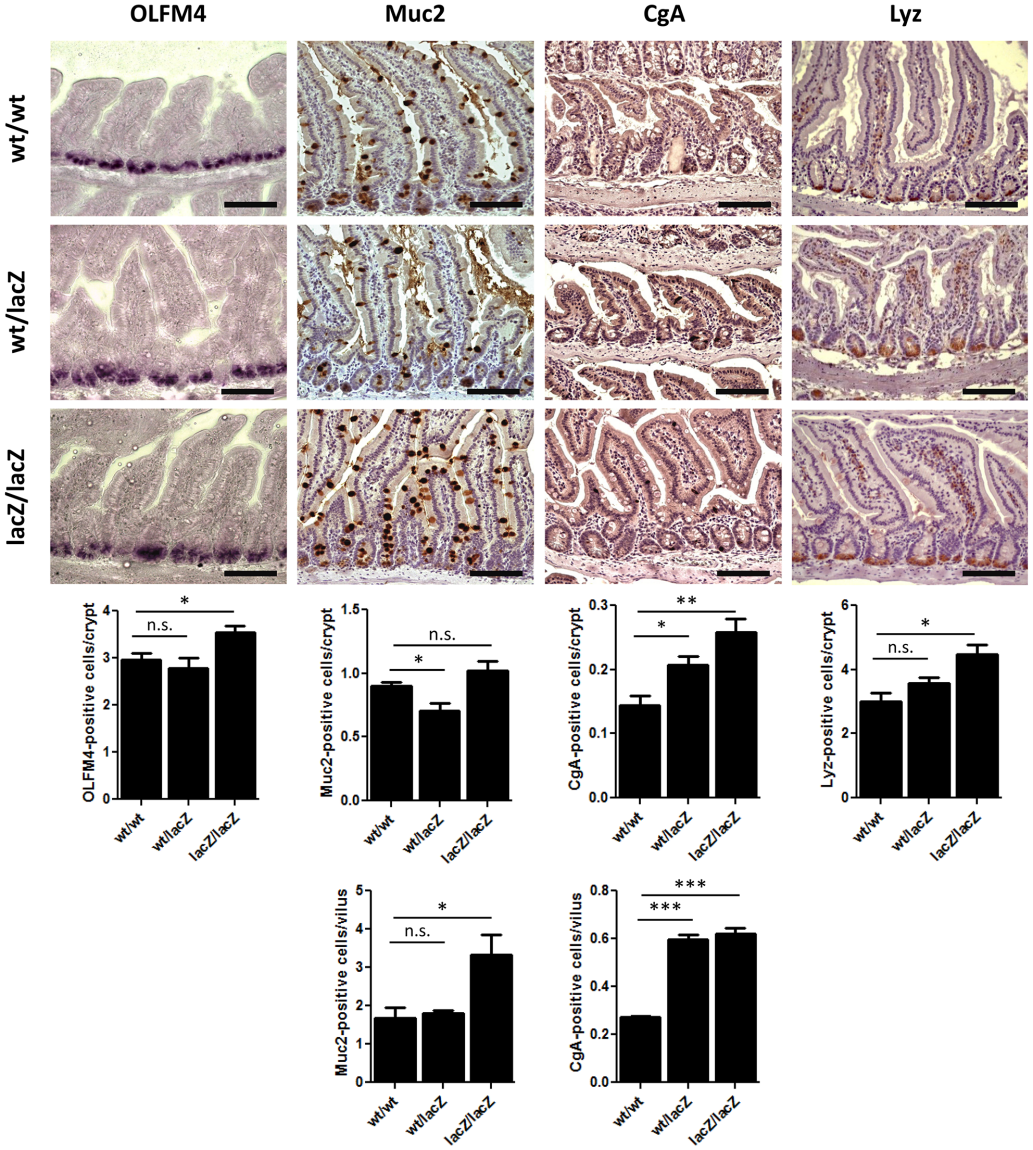
The loss of Usp22 results in a differentiation shift in the small intestine Reduction of Usp22 expression causes a slight increase in the number of olfactomedin 4 (OLFM4)-positive stem cells as revealed after *in situ* hybridization (wt/wt: *n* = 5; wt/lacZ: *n* = 4, *p* = 0.53; lacZ/lacZ: *n* = 5, *p* = 0.021). Immunohistochemistry shows an increase of differentiated cell types in the small intestine. The number of mucus-producing Goblet cells (Muc2) hardly varied in the crypts but was significantly increased in the villi (wt/wt: *n* = 5; wt/lacZ: *n* = 5, *p* = 0.69; lacZ/lacZ: *n* = 5, *p* = 0.023). A raised rate of enteroendocrine cells (CgA) was observed as well (wt/wt: *n* = 5; wt/lacZ: *n* = 5, *p* = 8.25.10–8; lacZ/lacZ: *n* = 5, *p* = 6.20.10–7). Paneth cells (Lyz) which are located at the bottom of the crypt show an increased presence in Usp22^lacZ/lacZ^ animals (wt/wt: *n* = 3; wt/lacZ: *n* = 3, *p* = 0.15; lacZ/lacZ: *n* = 3, *p* = 0.017). Scale bar: 100 μm.

We next assessed the effects of *Usp22* reduction on the cellular composition of the small intestine. Stem cells located near the bottom of crypts give rise to four types of differentiated cells in the small intestine: Goblet cells, enteroendocrine cells, Paneth cells and enterocytes, which account for the majority of cells in the intestinal epithelium. We therefore tested whether *Usp22* expression is required for the proper generation and distribution of differentiated cell types in villi and crypts. To assess the respective numbers of cell types, 4 month old mice were sacrificed and respective cell populations were examined via immunohistochemical staining (Figure 4). To determine whether the number of stem cells was affected by *Usp22* deficiency, we performed *in situ* hybridization analysis for the specific marker olfactomedin 4 (*Olfm4*) and observed a slight increase in the number of stem cells in the small intestines of Usp22^lacZ/lacZ^ animals. To determine whether this change had an effect on the respective proportions of differentiated cells, immunochemistry was conducted for Goblet, enteroendocrine and Paneth cells using respectively staining against the specific markers Mucin 2 (MUC2), Chromogranin A (CGA) and Lysozyme (LYZ). Goblet cells, responsible for mucus production in the small intestine, were counted in villi and crypts. The number of MUC2-positive cells in the crypts of Usp22^lacZ/lacZ^ mice did not significantly differ compared to Usp22^wt/wt^ mice. However, reduced levels of USP22 led to a nearly 2-fold increase in the abundance of Goblet cells in villi.

Staining for CGA to detect hormone-producing enteroendocrine cells revealed about 50% and 45% more enteroendocrine cells in the crypts and villi of Usp22^lacZ/lacZ^ animals, respectively. Paneth cells are located at the bottom of crypts. We found a clear increase of LYZ-positive cell frequency in the crypts of Usp22^lacZ/lacZ^ mice (four to five cells per crypt) compared to wild-type animals (three Paneth cells per crypt). Thus, while the reduction of *Usp22* expression did not affect the gross morphology of the small intestine, detailed analyses of the cellular composition suggests that proper intestinal epithelial cell differentiation in crypts and villi requires USP22 activity.

### *Usp22* is required for proper differentiation in the adult mouse brain

**Figure 5:**
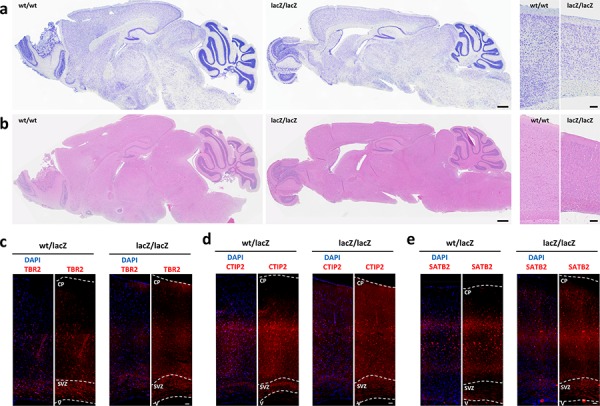
Loss of *Usp22* in adult mouse brain results in impaired cortical differentiation **a–b.** Usp22^lacZ^ animals at an age of 4 months have been assessed regarding to morphological changes of the different brain regions. Sagittal Nissl and H&E-stained sections revealed a less densely packed cortex in mice with USP22 reduction. Scale bar: 500 μm. **c–e.** Immunohistochemistry shows less differentiated cells and impaired layering in the adult cerebral cortex on coronal sections. The morphology and distribution of the subventricular zone (SVZ) with its intermediate precursor cells (TBr2) appears normal. The layering of the deep-layer (CTIP2, layer V–VI) and upper-layer neurons (SATB2, layer II–IV) is impaired. Scale bar: 100 μm. CP: cortical plate, SVZ: subventricular zone, V: ventricle.

To determine if the observed differentiation defect in *Usp22*-hypomorphic mice is restricted to the small intestine, or if this effect also occurs in other organs expressing high levels of *Usp22*, we examined the brains in adult Usp22^lacZ^ mice. We first investigated the overall effects of reduced *Usp22* expression on the brain morphology by using Nissl and H&E stained brain sections. We observed that the gross morphology of the adult was mainly unaffected, but the cerebral cortex was smaller and less densely packed in animals with reduced *Usp22* expression (Figure 5a–5b). This observation was in accordance with the expression pattern observed during forebrain development, which anticipated a potential cortical phenotype. We therefore analyzed further whether the loss of USP22 had an effect on the distribution of precursor or stem cells and differentiated neurons and on the layering of the cerebral cortex. We therefore examined the presence and number of progenitors of the subventricular zone as well as deep-layer and upper-layer neurons in adult wildtype and Usp22^lacZ/lacZ^ animals by immunohistochemistry. Intermediate precursor cells (IPC) of the subventricular zone were stained with an antibody specific for T-box transcription factor 2 (TBR2) also known as Eomes. The localization and distribution of the IPC in the subventricular zone was similar when comparing Usp22^wt/lacZ^ and Usp22^lacZ/lacZ^ animals (Figure 5c). Next, we determined whether, as seen in the intestinal epithelium, USP22 is required for the proper differentiation of glutamatergic neurons. Staining for a C2H2-type zinc finger protein (CTIP2) to detect differentiated early born deep-layer neurons and staining for special AT-rich sequence-binding protein 2 (SATB2) to mark later born upper-layer neurons revealed a specification defect in Usp22^lacZ/lacZ^ animals. Layer V-VI with the early born neurons appeared less densely packed, with fewer CTIP2 positive cells in Usp22^lacZ/lacZ^ compared to Usp22^wt/lacZ^ animals (Figure 5d). The later born neurons appeared more scattered within the cortical plate and were also less densely packed in USP22-depleted animals (Figure 5e). Taken together these results in the adult brain with the findings on the small intestine point at a role for USP22 in lineage specification and differentiation of stem or precursor cells in different organ systems.

### Effects of Usp22 knockout are independent of H2B monoubiquitination

**Figure 6:**
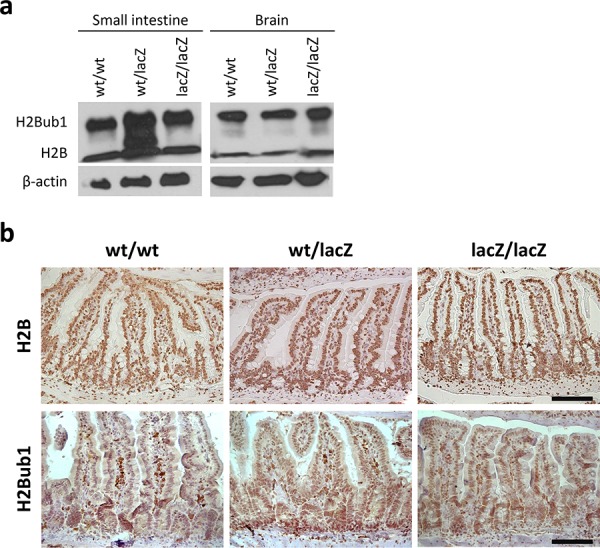
Differentiation defect is independent of H2B and H2Bub1 levels **a.** No difference in H2B and H2Bub1 levels was detected in protein samples isolated from Usp22^lacZ^ small intestines and brains. **b.** Immunohistochemical staining of H2B and H2Bub1 revealed that, regardless of the genotype, all cells are positively stained and show similar staining intensities (wt/wt: *n* = 4; wt/lacZ: *n* = 4; lacZ/lacZ: *n* = 4). Scale bar: 100 μm.

Previous studies revealed that Usp22 is able to affect transcription by the deubiquitination of histone H2B [12–14]. To clarify whether this function mediates the impairment in differentiation observed in our studies, we assessed the levels of H2Bub1 and H2B (as a control) in Usp22^lacZ^ mice. As revealed by Western blot analysis the global levels of H2Bub1 did not increase in the small intestine and brain of Usp22^lacZ^ mice (Figure 6a). To ensure that H2B and H2Bub1 are still equally distributed over the entire small intestine IHC was carried out. Based on the number of positive cells and the staining intensity no apparent differences in H2B and H2Bub1 levels were detected (Figure 6b).

## DISCUSSION

A previous study reported that the ablation of *Usp22* expression results in embryonic lethality at E10.5 of the post-implantation stage [22]. In contrast, here we describe a mouse model with a significant global reduction in *Usp22* levels which does not display embryonic lethality, but rather displays defects in cell fate determination in different tissues. The presence of LacZ and neomycin resistance cassettes downstream of the *Engrailed* splice acceptor site would be predicted to result in the termination of transcription. Our results show that Usp22^lacZ^ mice still show a minimal level of residual expression of normally spliced *Usp22* mRNA, which appears to be sufficient for the survival of these mice.

To date most studies related to *Usp22* function have focused on its potential role in promoting stem cell-like characteristics in diverse tumor types, including colorectal cancer. Indeed, our results suggest that decreased *Usp22* expression results in impaired differentiation in both the intestinal epithelium and in neurons in the cerebral cortex. Importantly, the role of USP22 during tumorigenesis is still largely unresolved. To date, most studies in patient samples were based either on microarray data or immunohistochemical analyses using antibodies whose specificity for USP22 is questionable. In our study we observe that, surprisingly, the gross morphology of the small intestine and the brain as well as the number of *Olfm*4-positive stem cells in *Usp22*-hypomorphic animals was largely unaffected, but rather a shift in the abundance of specific differentiated cell populations, i.e. Goblet and enteroendocrine cells, was increased or in post-mitotic neurons altered. This indicates that proper cell differentiation in the intestinal epithelium and the cerebral cortex is dependent on *Usp22* expression. However, whereas total cell numbers seem mostly unaffected in the small intestine, we observed fewer cells in the cerebral cortex. We conclude from this observation that although USP22 affects cell differentiation and/or lineage specification in general, its action might be under the control of organ specific cofactors responsible for context- or tissue-dependent phenotypes.

The results presented here provide important support that USP22 plays a central role in controlling lineage specification *in vivo*. Additional studies will be necessary to determine the molecular mechanisms by which it functions in these tissues. Our observations regarding differentiation could not be correlated to H2B and H2Bub1 levels suggesting that H2B is only partially deubiquitinated by Usp22. Moreover, a central question remains whether USP22 functions primarily as an epigenetic regulator, i.e., through its association with the SAGA complex, or whether other deubiquitination targets such as Sirt1 may be the central targets and mediators of USP22 function *in vivo*. Furthermore, tissue-specific ablation of *Usp22* alone or in the context of mouse tumor models will be essential for determining its function both in individual tissues as well as in the context of tumor formation and progression. Overall, the findings presented here further support an important biological function of *Usp22* and underscore the need for further *in vivo* and mechanistic studies to fully decipher its mechanisms of action and possible relevance as a therapeutic target in cancer.

## MATERIALS AND METHODS

### Generation of mice and genotyping

C57BL/6 embryonic stem cells expressing the LacZ gene under the control of the endogenous *Usp22* promoter were obtained from the University-Davis Knockout Mouse Project Repository (clone Usp22_D11). Stop codons and poly-A sites result in a reduced *Usp22* expression in these mice. Mice in this study were on the C57BL/6J background and used for analyses during embryonic stages or at an age to 3–4 months. Mice were genotyped by pre-heating PCR samples to 95°C for 3 min. The respective DNA fragments were amplified in 35 polymerization cycles with 95°C for 30 s, 60°C for 30 s, 72°C for 1 min. It was allowed for a final elongation at 72°C for 10 min (wt fw: 5′-GTGCCCTGGTTGCCCAGTGAG-3′;lacZ fw: 5′-CCCAGCTTTCTTGTACAAAGTGGTT-3′; wt/lacZ rv: 5′-CGGTTCAGGTGGATGCCGCA-3′).

### RNA isolation

Snap-frozen tissue material was homogenized in TRIzol (Invitrogen) and RNA was extracted according to the manufacturer's manual.

### Quantitative real-time PCR

mRNA was reverse-transcribed using random hexameric and oligo-dT primers. SYBR Green (Invitrogen/Life Technologies) was used for qRT-PCR analysis with following primer sets. 189F: 5′-GGAG CCTGAGGTCGAGGCCA-3′ and 359R: 5′-ACACAG GACTTTGCCTTGCGC-3′, 189F and 373R: 5′-CAGCC GGTTCAGGTGGATGCC-3′, 211F: 5′-AACCGGCTGC ACTCTTGCCT-3′ and 410R: 5′-TTCCAAGCCTTG CGCTGCTCC-3′, 932F: 5′-GCCAAGTCTGCCACGG GGTC-3′ and 1061R: 5′-GGTGGTCCCGGATGCAT GGC-3′.

### X-gal staining of tissue

Tissues were collected and fixed (6.75 ml 37% formaldehyde, 2 ml 25% glutaraldehyde, 5 ml NP-40, 25 ml 10x PBS, filled up to 250 ml with H_2_O, 110 mg DOC) on ice for 60 min. In the meanwhile, staining solution (2.5% 250 mM K_3_Fe(CN)_6_, 2.5% 250 mM K_4_Fe(CN)_6_, 2% 100 mM MgCl_2_ in PBS) was pre-warmed to 37°C and X-gal solution (40 mg Xgal/ml Dimethylformamide) was added. After washing in PBS, tissues were incubated in staining solution in the dark for 24 h. Embryos were frozen in TissueTek and cut on the sagittal plane. The frozen embryos were thawn in 0.1% PFA in PBS for approximately 10 min. Embryos were washed, dehydrated and prepared for paraffin embedding. After preparing 20 μm sections tissues have been counterstained using nuclear fast red-aluminum sulfate solution (Carl Roth).

### Histology

For H&E staining sections were de-paraffinized, rehydrated and stained in hematoxylin (Merck). After rinsing counterstaining with Eosin (Carl Roth) was carried out until a sufficient staining was obtained. Nissl staining of brain sections was performed in 0.5% cresyl violet for 10 min. The antigen retrieval for brain samples was performed by boiling sections in 0.2 M TBS pH 9.0. After blocking with 10% normal goat serum and 0.3% Triton X-100 in PBS, samples were incubated with primary antibodies in the blocking solution overnight at 4°C. The secondary antibodies were diluted in PBS and incubated for at least 1 h at RT. The following antibodies were used: Satb2 (Epitomics 2819-1, 1:100), Ctip2 (Abcam, ab18465, 1:200), Tbr2 (Millipore, Ab2283, 1:100). For immunohistochemical analysis of proteins in the small intestine sections antigen retrieval was performed by boiling slides in 10 mM citric acid/sodium-phosphate. Sections were quenched for endogenous peroxidases with 5% H_2_O_2_ in PBS and blocking was conducted using 10% fetal bovine serum (FBS) in PBS. Primary antibodies for Mucin 2 (Santa Cruz, sc-15334, 1:200), Lysozyme (Abcam, ab36362, 1:200), Chr-A (Santa Cruz, sc-13090, 1:200), H2B (Abcam, ab1790, 1:1000) and H2Bub1 ([7], 1:20) were diluted in PBS containing 10% FBS and incubated overnight at 4°C. Biotinylated secondary antibodies were diluted 1:200. Sections were incubated with ExtrAvidin-Peroxidase (Sigma) diluted 1:1000 in PBS the staining was developed using DAB (Immpact™ DAB Peroxidase Substrate Kit SK-4105 [Vector]). Hematoxylin was used for counterstaining. 100–200 crypts or villi were counted per small intestine section obtained from at least 4 mice per genotype.

### *In situ* hybridization

pBlue *Olfm*4 plasmid has been linearized overnight. For the *in vitro* transcription using a DIG RNA labeling kit (Roche), two reactions have been set up each with 2 μg of linearized plasmid DNA. Mixtures have been incubated at 37°C for 4 h and RNA was subsequently purified. An equal volume of formamide was added and stored at −20°C. 9 μm thick paraffin sections of the small intestine have been deparaffinized and rehydrated. Slides were incubated in 0.2 N HCl for 15 min. After rinsing with DEPC-treated water, sections were treated with Proteinase K (Invitrogen) in PBS for 20 min which has been pre-warmed to 37°C. Slides were rinsed in 0.2% glycine in PBS and PBS to stop the reaction. Sections have been post-fixed in 4% PFA for 10 min. Slides were incubated in PBS and 5x SSC buffer (750 mM NaCl, 75 mM Na_3_C_6_H_5_O_7_.2H_2_O in H_2_O) for 5 min each. Sections were pre-hybridized by adding 400 μl hybridization solution per slide and stored at 68°C h in a humid chamber containing a 5x SSC/formamide mixture (1:1) for 1 h. Prehybridization solution was replaced with hybridization solution containing 500 ng/ml DIG-labelled OLFM4 RNA probe. Slides were incubated at 68°C for approximately 90 h.

Slides were rinsed twice in 2x SSC (pH 4.5) and washed in triplicate with 50% formamide/2x SSC (pH 4.5) at 63°C for 20 min. After rinsing in 5x TBS-T, slides were incubated in blocking solution (0.5% blocking powder [Roche] in TBS-T) at room temperature for 2 h. Anti-DIG Fab (Roche, 11093274910) was diluted 1:2000 in blocking solution and incubated at 4°C overnight. Slides were rinsed five times in TBS-T and three times in NTM buffer (100 mM NaCl, 100 mM Tris, 50 mM MgCl_2_, in H_2_O). Staining was developed by incubation in NBT/BCIP solution (10 ml NTM buffer, 333 μl NBT solution (10 mg tablet dissolved in 1 ml H_2_O), 35 μl BCIP (25 mg tablet dissolved in 500 μl dimethylformamide), 25 μl 1 M levamisole) until blue staining could be observed. Subsequently slides were rinsed in NTM buffer, dehydrated and embedded as described above. Positive cells localized in 100–200 crypts have been counted.

### Western blot

For Western blot analysis brains and small intestines were lysed in RIPA buffer (1% NP-40, 0.5% sodium deoxycholate and 0.1% SDS in PBS) containing 1 mm N-ethylmaleimide, 1 mm Pefabloc and 1 μg/ml Aprotinin/Leupeptin. Proteins were separated by SDS–polyacrylamide gel electrophoresis and transferred to a nitrocellulose membrane. Proteins were detected using antibodies against Usp22 (Santa Cruz, sc-390585), H2B (Abcam, ab1790), H2Bub1 ([7]), GAPDH (Abcam, ab8245) and β-actin (Abcam, ab8227) and horseradish peroxidase-conjugated secondary antibodies (Santa Cruz).

### Statistical analyses

All graphs in this study have been designed with GraphPad Prism version 5.04 (GraphPad Software, Inc.). *P*-values were determined using Student's *t*-test.
